# Monolithic 3D Printing of Origami‐Inspired Soft Robotics from Sustainable Bio‐Based Resin

**DOI:** 10.1002/advs.202520529

**Published:** 2026-01-21

**Authors:** Ramin Montazeri, Hugo de Souza Oliveira, Xin Li, Qingchuan Song, Bastian E. Rapp, Dorothea Helmer, Edoardo Milana

**Affiliations:** ^1^ Laboratory of Process Technology NeptunLab Department of Microsystems Engineering (IMTEK) University of Freiburg Freiburg Germany; ^2^ Freiburg Materials Research Center (FMF) University of Freiburg Freiburg Germany; ^3^ Laboratory for Soft Machines SoftLab Department of Microsystems Engineering (IMTEK) University of Freiburg Freiburg Germany; ^4^ Cluster of Excellence livMatS @ FIT – Freiburg Center For Interactive Materials and Bioinspired Technologies University of Freiburg Freiburg Germany

**Keywords:** bio‐based materials, high resolution 3D printing, origami actuators, soft gripper, soft robotics, sustainable materials

## Abstract

Soft robotics has gained significant attention for its potential to deliver safe, adaptable, and biocompatible machines, by embracing the mechanical compliance of soft materials. However, the manufacture of soft robotic devices and machines still largely relies on petroleum‐based polymers. Furthermore, in light‐induced 3D printing, a key technology for fabricating complex 3D monolithic soft robots, non‐sustainable resins remain predominant. This work addresses this issue by developing a photocurable bio‐based resin to monolithically fabricate soft robots. We formulate a resin using soybean oil as a renewable precursor and shape it via Digital Light Processing into an origami‐inspired vacuum‐actuated actuator. The bio‐based material has a Young's modulus of 18.9 MPa and an elongation at break of 19.6%. The origami deformation, based on folding rather than stretching, enables actuator operation, despite the lower elongation range of our material compared to silicone elastomers. We report on the characterization of the bulk material properties and the mechanical performance of the actuator, which performs 2000 cycles without failure before testing ceased. Finally, we design and fabricate a monolithic soft robotic gripper with integrated origami actuation using our bio‐based material. We show the functional operation of the gripper in grasping different objects, as well as in underwater settings.

## Graphical abstract

This work develops a photocurable, bio‐based resin from soybean oil for the monolithic fabrication of sustainable soft robots via Digital Light Processing. An origami‐inspired, vacuum‐actuated soft gripper is designed and fabricated, enabling reliable operation despite the material's lower elongation. The origami structure withstands 2000 cycles, and the gripper demonstrates versatile grasping of objects with varying stiffness, including underwater applications.

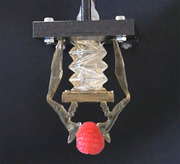

## 1 Introduction

Soft robots have attracted a lot of attention lately due to their inherent elasticity, making them safe and adaptive when interacting with the environment [1]. They are designed and fabricated exploring materials with mechanical properties that are several orders of magnitude lower than the materials used in traditional robots [2]. This allows for a safer interaction with more delicate structures and objects, such as human organs and fragile environments. Additionally, they can be designed to exhibit a physical behavior that is encoded to their structure, reducing the need for external microcontrollers [3].

Fluidically‐driven soft actuators are very common in soft robotics, due to their large forces, monolithic designs and design space [4]. To realize fluid‐driven soft robots, various design approaches have been explored, such as pneumatic networks (PneuNet) [5], bellows [6], fiber‐reinforcement [7], chamber asymmetries [8], balloons [9], and shells [10]. However, all these structures experience significant stretching under applied internal pressure. In contrast, origami‐based designs [11] do not suffer from this issue, as their structures fold and unfold when pressure changes, offering significant structural adjustments with less amount of elastic energy stored. Additionally, they offer compactness and portability, as they can be folded and deployed in confined spaces. The folding pattern can be scaled to different sizes, allowing for the creation of macro [12] and micro [13] structures. For those reasons, origami has been utilized to create soft actuators and sensors for soft robotics. Various origami folding mechanisms, including Kresling, Miura, Yoshimura, and Accordion, etc. have been investigated to develop different types of actuators [14] and sensing [15] mechanisms.

Currently, origami‐inspired soft robotics (OISRs) faces two pressing challenges: material sustainability and fabrication complexity. As an emerging field, soft robotics in general and OISRs in particular, holds the potential to embed sustainability principles at the early stage. Unlike more established technologies that must undergo costly transitions toward greener practices, soft robotics can integrate eco‐friendly design, material selection, and manufacturing processes already at the research and development level [16, 17, 18]. This approach has been exemplified by the development of a bio‐based elastomer for the fabrication of a PneuNet‐based soft gripper [19]. However, in the context of origami soft structures, despite recent progress toward biodegradable [20], biocompatible, [21, 22] and bio‐based materials [23] (none of which are fluidically driven), most reported systems still rely predominantly on conventional petroleum‐based elastomers, such as silicone‐based materials and thermoplastic polyurethane (TPU). This widespread reliance is associated with sustainability concerns related to environmental footprint, urging the need for the development of more sustainable material platforms. Moreover, the lack of scalable, monolithic fabrication strategies constrains the realization of complex 3D OISRs with enclosed cavity, needed for fluidically‐driven actuation. Conventional approaches, including folding‐based gluing assembly [20, 24] and multi‐step casting or molding [25, 26], not only impose inherent limitations on structural complexity but also are labor‐intensive and time‐consuming.

Recent advances in 3D printing are radically changing OISR fabrication, offering significant advantages over conventional methods. Among these benefits are the mold‐free, rapid prototyping of high‐resolution, complex structures while minimizing material waste [14, 21, 22], thereby aligning with sustainability objectives. Extrusion‐based methods such as fused deposition modeling (FDM) and direct ink writing (DIW) have been most widely applied for fabrication of OISRs due to their low cost and accessibility. For instance, tubular origami‐inspired structures with heat‐activated shape memory were fabricated using flexible polylactic acid (PLA) filaments via FDM [22]. In another approach, electrothermally controlled origami was produced from PLA and continuous carbon fiber using a dual‐nozzle FDM 3D printer [21]. Additionally, flexible hinge‐based origami structures were manufactured from silicone via DIW [27]. Some of these structures were realized by a single‐step monolithic printing approach employing a gel support, while others require a multi‐step print–assemble–glue process. Such cumbersome multi‐step assemblies prevent the realization of truly monolithic OISR systems. Additionally, extrusion‐based 3D printing generally suffers from limited resolution and difficulty in producing leak‐proof inflatable structures [28].

To address the limitations of extrusion‐based methods, light‐induced 3D printing techniques, notably digital light processing (DLP) and stereolithography (SLA), have gained prominence. These techniques exploit photopolymerization to achieve high resolution, small feature sizes, and complex geometries inaccessible to extrusion‐based 3D printing. However, most reported applications of light‐induced 3D printing for OISRs have focused on the fabrication of planar or quasi‐2D origami structures, which are subsequently transformed into relatively simple 3D configurations in response to external stimuli such as heat, light, or pH. Examples include the fabrication of reconfigurable origami structures consisting of rigid polymer panels and shape memory hinges derived from polymer precursors, such as isobornyl acrylate (roughly 60 wt.%) using DLP [23]. These 2D patterns were transformed into 3D structures either temporarily, through shape‐memory behavior programmed via thermomechanical cycling, or permanently, through reconfiguration and weldability enabled by bond exchange reactions at elevated temperatures. The optimized resin for DLP 3D printing exhibited a printing resolution of 100 µm, a Young's modulus of 1.06 GPa at 25°C, a strain at break of 50% at 25°C, and an ultimate tensile strength of 30 MPa at 25°C. The force required to fully open the folded origami structure was 399 N at 25°C. In another study, multilateral origami sheets capable of being programmed into 3D forms were fabricated using a DLP‐based method [29]. In this case, the rigid panels and shape memory polymer hinges were 3D printed from non‐sustainable materials, including tert‐butyl acrylate and aliphatic urethane diacrylate. In a related approach, planar origami sheets were produced through DLP using non‐sustainable resin containing n‐butyl acrylate, a volatile monomer [30]. Its volatilization upon post‐print heating induced volume shrinkage, thereby enabling the transformation of the origami structures from 2D to 3D forms.

Although these studies demonstrate the potential of light‐induced 3D printing for origami‐inspired actuators, they neither achieve monolithic fabrication of fluidically driven OISRs nor overcome the heavy reliance on petroleum‐derived resins, leaving both fluidic actuation capabilities and sustainability largely unaddressed. In terms of fluidic actuation, a recent study reported a bio‐based resin from rapeseed oil acrylate and isobornyl acrylate for DLP fabrication of a pneumatically actuated soft gripper [19], although the reported system did not incorporate inflatable origami structures. While several formulations with a bio‐based carbon content (BCC) ranging from 62% to 80% were developed, the final device was fabricated using the resin with the lowest BCC (62%, 70 wt.% renewable) due to functional limitations, while more sustainable formulations proved unsuitable. The resin employed for the fabrication of the flexible actuators exhibited a printing resolution of approximately 200 µm, a Young's modulus of 3.6 MPa, an ultimate tensile strength of 4.3 MPa, and a strain at break of 137%. Moreover, the gripper was not fabricated monolithically but manually assembled, still relying on discrete component assembly and varied post‐processing.

Taken together, despite recent progress in combining DLP 3D printing with bio‐based resins, no existing approach has successfully addressed this critical gap in the field: the need for monolithic fabrication of inflatable 3D OISRs using renewable, environmentally benign feedstocks.

This study addresses this critical gap by exploring the synergistic integration of a highly sustainable bio‐based resin with DLP for the monolithic fabrication of a fluidically actuated origami‐driven soft gripper. To achieve this, we develop a 3D origami contracting actuator based on Kresling origami pattern that can be actuated by applying negative pressure as control input. Further, we design the monolithic soft gripper, consisting of a compliant mechanism driven by the origami actuator. To ensure an enhanced sustainability, we formulate a DLP resin based on acrylated epoxidized soybean oil (AESO) and tetrahydrofurfuryl methacrylate (THFMA) for monolithic fabrication of this design. The developed resin comprises 90 wt.% renewable components and achieves a BCC of 73%.

We demonstrated, for the first time, the monolithic fabrication of an origami‐inflatable soft robotic gripper in a single DLP printing step, eliminating the need for multi‐step assembly, folding, or post‐print transformation. The gripper's potential for manipulating delicate soft structures is also shown.

## 2 Results and Discussion

### 2.1 Design of the Origami‐Inspired Actuator and the Soft Gripper

**FIGURE 1:**
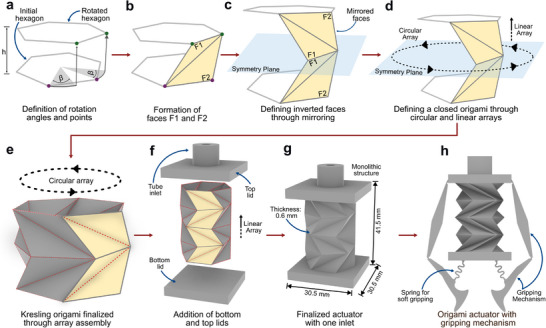
Design and assembly of a Kresling‐based origami actuator: (a) Definition of the initial hexagonal geometry, rotation angle (β), and height (h). (b) Formation of the primary triangular faces (F1 and F2). (c) Creation of an inverted unit by mirroring the faces across a symmetry plane to compensate for rotational torsion. (d) Conceptualization of the circular and linear arraying process to form a closed structure. (e) The resulting origami pattern after arraying. (f) Exploded view showing the addition of top and bottom lids to create a sealed chamber with a single pressure port. (g) The final, monolithic actuator design. (h) Integration of the actuator with a compliant gripping mechanism to create the final soft gripper.

Figure 1 illustrates the design process of the origami contracting actuator and the soft gripper. Among various origami‐inspired mechanisms reported in the literature, including Miura, Yoshimura, Accordion, and Waterbomb patterns, the Kresling geometry was selected due to its suitability for monolithic, sealed, pneumatic soft robotic actuation, following design strategies for monolithic origami fabrication previously demonstrated in the literature [31]. Comparative studies on compliant and additively manufactured origami tubes have shown that Yoshimura‐based structures often deform through global buckling rather than controlled folding, leading to less predictable kinematics, while Accordion and Waterbomb patterns are typically optimized for pure axial compression or radial expansion and do not naturally form continuously sealed tubular geometries suitable for inflatable actuators [22, 32]. Miura‐ori–based designs are primarily intended for planar or open‐surface transformations and therefore present additional challenges for pressure sealing in pneumatic systems [32].

In contrast, the Kresling pattern forms a continuous closed tubular surface and deforms predominantly through folding at predefined crease lines, enabling predictable axial contraction [22, 33]. By combining pairs of Kresling patterns with their mirrored counterparts, it is possible to preserve the predictable folding behavior and fabrication advantages of the Kresling pattern while eliminating undesired rotational motion, making the structure particularly suitable for linear actuation in soft robotic grippers.

Following this design choice, the actuator geometry is constructed using a parametric approach. The design process begins with the definition of an initial hexagon with two points located on one of its sides (Figure 1a). These two points are rotated with respect to the center of the hexagon by an angle *β*, as demonstrated by the purple point in Figure 1a. Next, the points are elevated by a distance *h*, which corresponds to the height of the Kresling pattern, forming the side of a rotated hexagon, as shown by the green points in Figure 1a. The green and purple points are connected to form the first two faces of the Kresling structure, F1 and F2, as illustrated in Figure 1b. A symmetry plane parallel to the initial hexagon and located at a distance *h* is then used as a reference to mirror the faces F1 and F2, generating the base unit of the inverted Kresling origami. The enclosed structure is obtained by applying a circular array followed by a linear array, as shown in Figure 1d, resulting in the finalized origami actuator geometry presented in Figure 1e.

To enable pressure‐driven actuation, the upper and bottom parts of the origami structure are closed, leaving a single pneumatic outlet. For this purpose, a bottom lid and a top lid with a tubular outlet are added, as illustrated in Figure 1f. The finalized monolithic actuator, shown in Figure 1g, measures 30.5 mm × 30.5 mm × 41.5 mm and features an origami face thickness of 0.6 mm, a rotation angle *β* of 75°, and a height of *h* of 8.23 mm. Finally, the soft gripper is obtained by combining the origami actuator with a compliant gripping mechanism, which translates the linear contraction of the origami structure into the closing motion of the gripper (Figure 1h).

While the present study focuses on a Kresling‐based architecture as a representative example, this approach can, in principle, be extended to other origami‐inspired geometries based on continuous surfaces and bending‐dominated deformation, provided that constraints related to feature resolution, crease thickness, and pressure sealing are satisfied by the process technology. These conditions define the current scope and generalizability of the proposed fabrication strategy.

### 2.2 Resin Development and Fabrication Process

A biobased resin from renewable sources is developed for monolithic printing of the origami gripper. Acrylated epoxidized soybean oil (AESO) is chosen as the resin base due to its renewable origin, inherent biocompatibility, readily functionalizable unsaturated double bonds, and cost‐effective availability. For optimal printability in light‐induced 3D printing, it is essential to regulate the viscosity of the resin, as this property governs its flow behavior within the vat. Due to the inherently high viscosity of AESO, either tetrahydrofurfuryl acrylate (THFA) or tetrahydrofurfuryl methacrylate (THFMA), derived from hemicellulose, is introduced as a bio‐based reactive diluent to improve rheological properties for printing. Either pentaerythritol tetraacrylate (PETA) or Genomer 4230 is incorporated as a cross‐linker to enhance mechanical integrity, while Tinuvin 326 functioned as a UV absorber to balance light penetration during printing. Several preliminary formulations are investigated even though they could not be suitable for reliable printing. Their compositions are summarized in Table S1, along with a concise discussion of the factors that limited their performance. The initial formulation (Resin 1) suffers from insufficient mechanical strength, which can be attributed to the relatively low cross‐link density. To overcome this limitation, PETA, a tetrafunctional cross‐linker, is introduced in Resin 2 to increase the number of covalent junction points within the polymer network, thereby enhancing its mechanical robustness. However, to further optimize the balance between strength and flexibility, PETA is subsequently replaced with the difunctional cross‐linker Genomer 4230 in Resin 3. The reduced functionality of Genomer 4230 lowers the cross‐link density. Finally, to further improve printability, THFMA is employed in place of THFA in Resin 4, ultimately yielding a suitable resin for 3D printing. The optimized resin formulation consists of 75 wt.% AESO, 15 wt.% THFMA, 9.5 wt.% Genomer 4230, and 0.5 wt.% Tinuvin 326, with 0.5 wt.% diphenyl(2,4,6‐trimethylbenzoyl)phosphine oxide (TPO) added as photoinitiator (relative to the total mass of the other components).

**FIGURE 2:**
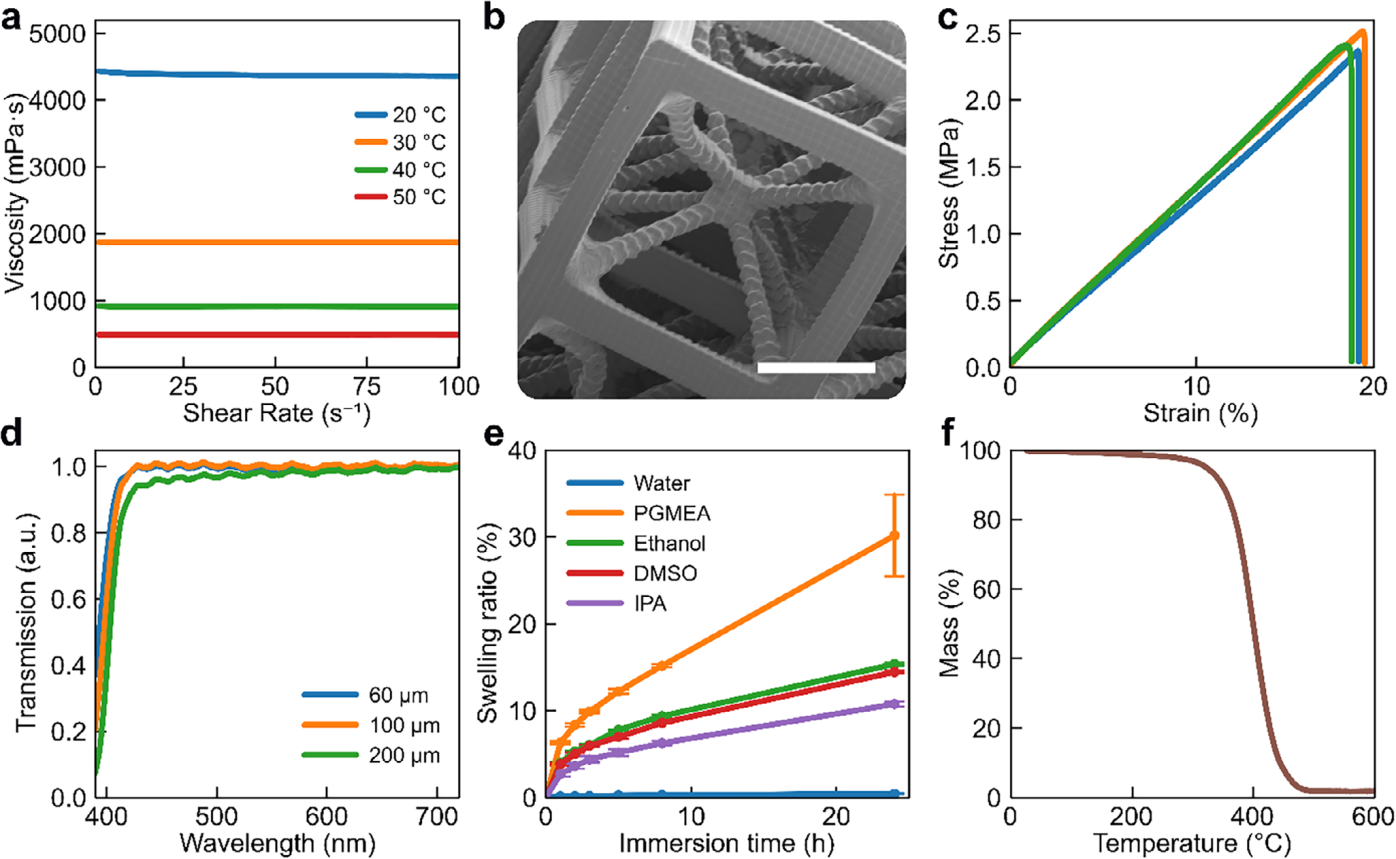
Physicochemical, mechanical, microscopic, and thermal characterization of the bio‐based resin for light‐induced 3D printing. a) Shear rate‐dependent viscosity at different temperatures, decreasing from 4377 mPa·s at 20°C to 492 mPa·s at 50°C, remaining well below the 10 Pa·s threshold for DLP resins and enabling ambient‐temperature 3D printing. b) Scanning Electron Microscopy (SEM) imaging of a cubic lattice test structure fabricated via DLP, demonstrating successful 3D printing of microscale features with dimensions down to roughly 90 µm (scale bar: 500 µm). c) Stress–strain response of vertically printed ISO 527‐2 type 5B specimens, showing linear elasticity without plastic deformation; the Young's modulus, ultimate tensile strength, and strain at break were 18.9 ± 0.5, 2.4 ± 0.1, and 19.6 ± 0.3%, respectively. d) UV–vis transmission of cured films (60 – 200 µm) exhibiting >97% transparency at 550 nm with slight thickness‐dependent attenuation. e) Swelling behavior after 24 h in water and certain solvents, demonstrating negligible uptake in water, moderate swelling in polar alcohols and DMSO (10 – 15 wt.%), pronounced swelling and cracking in PGMEA (ca. 30 wt.%), and disintegration in acetone. f) Thermogravimetric analysis (TGA) of 3D printed specimen showing a uniform, single‐stage degradation profile typical of cross‐linked networks, with an onset degradation temperature (T_5_) of 321°C, maximum decomposition rate at 393°C, and <2 wt.% char yield at 500°C.

To evaluate the renewable characteristic of the resin and its suitability as sustainable functional materials in soft robotics, the BCC is determined. The final resin exhibits a BCC of 73%, reflecting its high renewable fraction and alignment with the development of bio‐based functional materials. To ensure that the final resin meets the viscosity criteria for DLP resin printing, which is a viscosity below 10 Pa·s [34], the viscosity of the resin is analyzed. As shown in Figure 2a, the viscosity decreases progressively with increasing temperature, from 4377 mPa·s at 20°C to 492 mPa·s at 50°C, respectively. This indicates that printing can be performed under ambient conditions without thermal assistance. To enable the monolithic fabrication of the soft gripper's features via DLP, printing parameters are systematically adjusted in accordance with the resin's reactivity, light penetration characteristics, viscosity, and the geometrical complexity of the targeted structures. The optimized printing parameters for the soft gripper are set to 40 µm layer thickness, 3 s exposure time, and 25 mW cm^−^^2^ light intensity. After printing, unreacted resin was removed using isopropanol (IPA). The monolithic origami‐inspired soft gripper was successfully fabricated with the biobased resin via high resolution DLP (Figure S1). To evaluate the 3D printing resolution, a cubic lattice structure is fabricated via DLP. Scanning Electron Microscopy (SEM) imaging (Figure 2b) confirms the realization of microscale features with dimensions down to roughly 90 µm. This achieved minimum feature size is approximately twice the pixel resolution of the 3D printer, demonstrating the resin's capability for high‐resolution 3D printing.

### 2.3 Material Characterization

Tensile tests are conducted on vertically printed specimens (ISO 527‐2 type 5B) to assess intrinsic material behavior. During testing, the tensile load is applied perpendicular to the printed layers. Stress–strain curves (Figure 2c) reveal a linear relationship with no plastic deformation, indicating an exclusively elastic response and limited energy dissipation prior to fracture. The measured Young's modulus, ultimate tensile strength, and strain at break are 18.9 MPa ± 0.5 MPa, 2.4 MPa ± 0.1 MPa, and 19.6% ± 0.3%, respectively. Although the intrinsic elongation at break of the material is modest compared to the elastomers typically used in soft robotics, the origami‐inspired geometry of our soft robotic devices imparts structural flexibility and geometrical compliance, thereby extending the applicability of materials with limited elongations in soft robotics. The optical transmission of the cured resin is analyzed by UV–vis spectroscopy (Figure 2d). Optical clarity of resins broadens their applicability in soft robotics, particularly in scenarios where transparency is beneficial, such as visual inspection, light‐based sensing, and aesthetic integration. Samples with thicknesses of 60 µm, 100 µm, and 200 µm consistently exhibit high transparency with a transmission exceeding 97% at 550 nm. The chemical resistance and structural integrity of the bio‐based 3D‐printed materials were evaluated by measuring swelling behavior in water and selected organic solvents (Figure 2e). After 24 h of immersion in water, the mass swelling ratio was negligible, confirming the hydrophobic character of the soybean oil‐derived network. Moderate swelling was observed in polar organic solvents, with mass increases of approximately 10 wt.% in IPA and about 15 wt.% in both ethanol and dimethyl sulfoxide (DMSO), while the specimens retained their structural integrity. In contrast, immersion in propylene glycol monomethyl ether acetate (PGMEA) induced more pronounced swelling (ca. 30%) and crack formation in all samples, which accounts for the larger error bars at 24 h. Acetone exposure resulted in catastrophic failure, with complete disintegration of the specimens within 3 h due to extensive cracking. Collectively, these findings demonstrate that the 3D‐printed bio‐based material exhibits chemical stability in aqueous environments and tolerates short‐term exposure to common alcohols and DMSO, thereby underscoring its potential suitability for soft robotic grippers operating in water‐rich or bio‐relevant environments. Thermogravimetric analysis (TGA) was conducted to determine the degradation temperature and thermal stability of the printed materials. The TGA thermogram (Figure 2f) revealed a uniform, single‐stage degradation profile, characteristic of cross‐linked networks. The 3D‐printed specimens exhibited an onset degradation temperature (T_5_, 5% weight loss) of 321°C and a maximum decomposition rate at 393°C. The char yield was below 2 wt.% at 500°C, indicating nearly complete volatilization of the organic matrix, consistent with the aliphatic triglyceride backbone of AESO. The relatively high degradation onset confirms that the printed materials remain stable within the operational temperature range of soft robotic applications, where thermal loads rarely exceed 100°C. These results demonstrate that the bio‐based resin combines sustainability with sufficient thermal robustness for functional deployment in soft robotic grippers.

### 2.4 Origami‐Inspired Contracting Actuator

**FIGURE 3:**
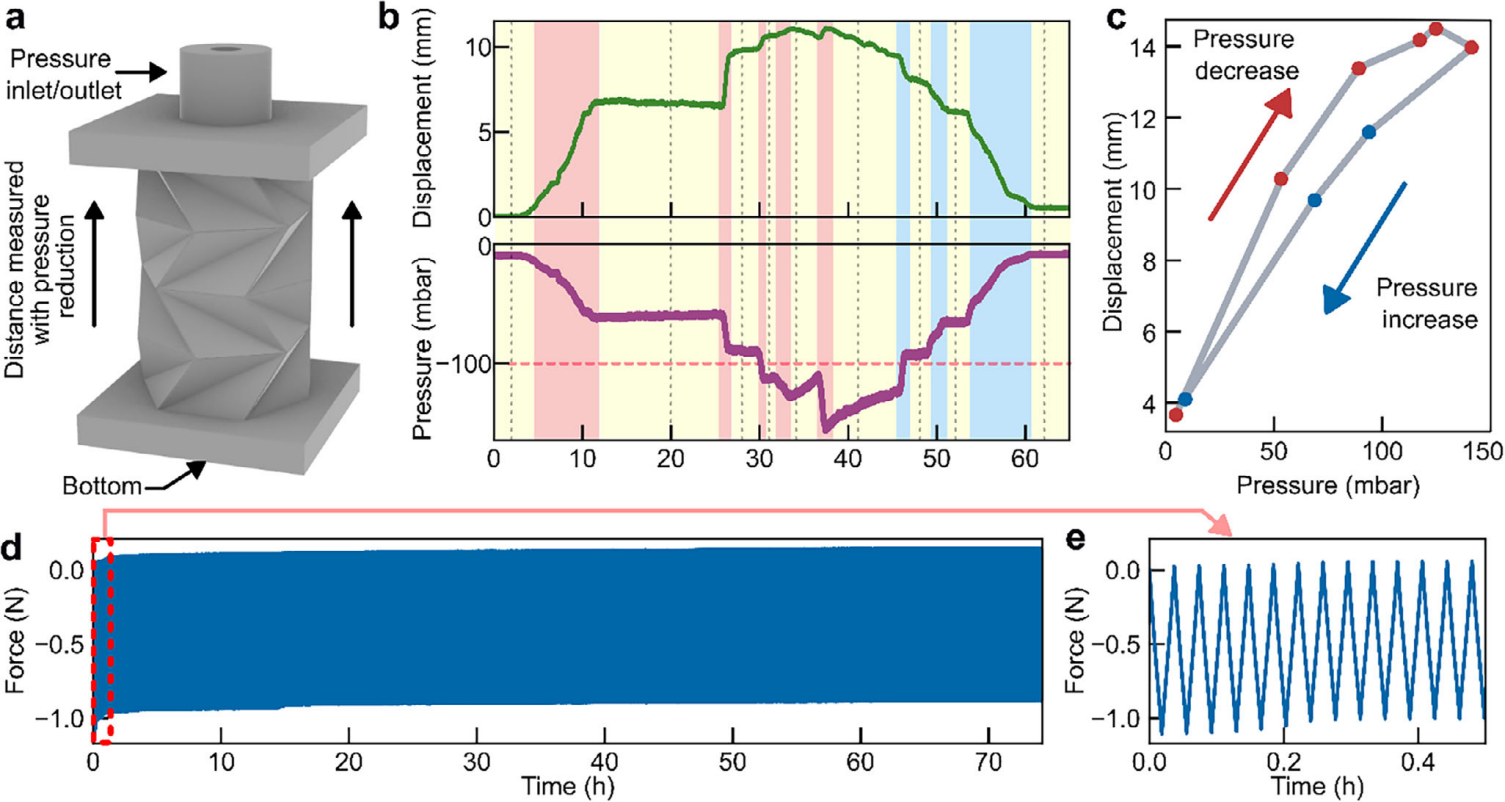
Mechanical Characterization of the Origami Actuator. (a) Schematic illustration of the actuator setup for pressure‐displacement characterization. (b) Actuator displacement and internal pressure as a function of time, highlighting periods of valve opening (red), valve opened (yellow), and valve closed (blue). (c) Pressure‐displacement hysteresis loop derived from representative points in (b), illustrating energy dissipation. (d) Long‐term cyclic compression test of the actuator over 70 h. (e) Magnified view of the initial cycles from (d), showing the stabilization of the mechanical response within the first 30 min of test.

The mechanical performance of the actuator is characterized by connecting its pressure outlet to a manually controlled negative‐pressure source, as illustrated in Figure 3a and detailed in Figure S2, through a valve that regulated the airflow. When the valve is opened, air inside the actuator is drawn toward the negative‐pressure reservoir, reducing the internal pressure and causing the origami structure to contract axially.

Figure 3b shows the relation between internal pressure and the corresponding axial displacement as a function of time. The red, yellow, and blue shaded regions correspond to distinct valve states. In the red regions, the valve is progressively opened, producing a decrease in internal pressure and a linear displacement of the origami; in the yellow regions, the valve position is kept fixed, resulting in nearly steady pressure; and in the blue regions, the valve is closing, allowing the internal pressure to return to ambient pressure.

For pressure levels above −100 mbar (e.g., between 12 and 26 s), the actuator maintains a stable internal pressure and constant axial contraction, indicating an equilibrium state. However, when the negative pressure dropped below −100 mbar (e.g., between 38 and 46 s), the actuator initially contracts as expected but does not sustain a constant displacement. Despite the valve position remaining fixed, the internal pressure gradually increases over time.

This pressure recovery is attributed primarily to air leakage through manufacturing‐induced defects, such as interlayer gaps or locally unsealed regions in the 3D‐printed origami structure. When the pressure differential becomes sufficiently large, these defects allow ambient air to infiltrate the cavity, partially compensating for the imposed vacuum. The pressure–displacement relationship shown in Figure 3c displays a characteristic hysteresis loop, originating from both viscoelastic energy dissipation in the polymer and pneumatic losses associated with transient airflow through these leakage paths. A quantitative leakage analysis based on pressure–time data at fixed setpoints is provided in Figure S5, which shows that under moderate deformation levels, the leakage rate remains low (around 0.25 mbar s^−^^1^), whereas at larger deformations and more negative pressures the leakage rate increases significantly, reaching 5.2 mbar s^−^^1^.

Mitigation strategies to reduce leakage in the structure involve both controlling the operational deformation regime and minimizing manufacturing‐induced defects associated with the DLP printing process. Maintaining operation within defined deformation limits reduces stress localization at the crease regions, while high printing quality (e.g. precise control of layer thickness and exposure in regions critical for sealing) improves interlayer continuity. In addition, optimized resin flow during printing, achieved through appropriate dwell times and peel velocities, together with geometric optimization of crease geometry and face thickness, can further reduce wall‐thickness variations, air entrapment, and micro‐gaps, thereby improving pressure retention.

To evaluate the actuator's longevity under representative operating conditions, a fatigue test is performed. The structure is mounted in a tensile testing machine and subjected to 2000 compression–extension cycles at a deformation rate of 0.1 mm s^−^^1^, corresponding to a total testing time exceeding 70 h. During this test, the actuator is cyclically deformed to 20% of its initial height, a deformation level that remains well above the axial deformation required for functional operation of the gripper (Section 2.5). The maximum force measured during the test is around – 1 N, shown in Figure 3d. The peak and trough forces stabilize after approximately 50 min, demonstrating a consistent mechanical response for the remainder of the test. Figure 3e provides a magnified view of these initial cycles where this stabilization occurs. Throughout the extended fatigue test, the actuator exhibits stable mechanical behavior without catastrophic failure.

Failure mechanisms are investigated separately under accelerated loading conditions. When subjected to rapid cyclic compression beyond 30% deformation, failure consistently initiates as micro‐cracks along the origami crease lines, which experience localized stress concentration during cyclic folding. These cracks progressively propagate with continued deformation until eventual rupture. A detailed analysis of the fatigue behavior and failure modes is provided in Figure S6.

Table S2 presents a quantitative comparison between the present work and two representative, closely related studies, highlighting recent advances in sustainable DLP‐based 3D printing of soft actuators.

### 2.5 Soft Gripper

**FIGURE 4:**
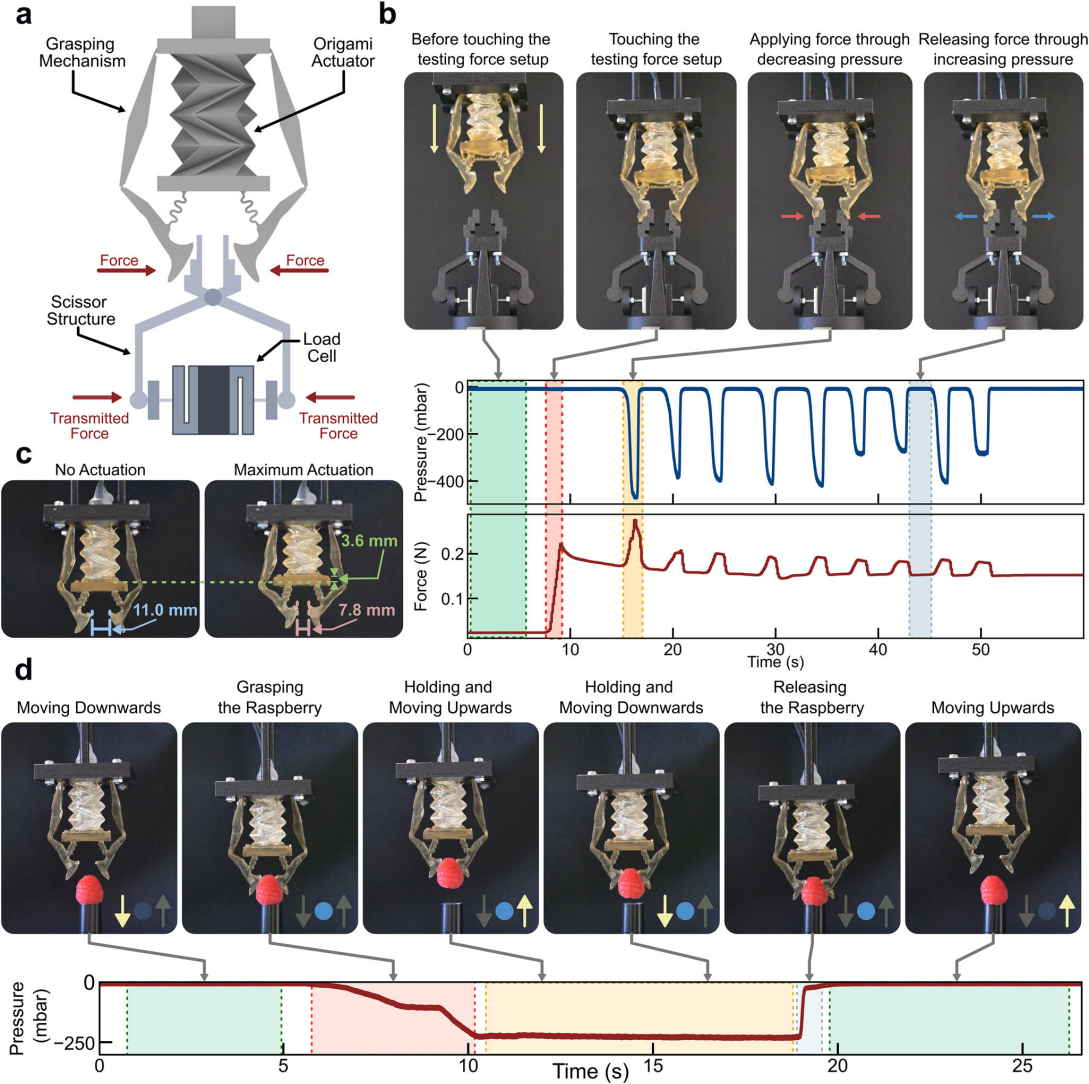
Gripper Performance and Delicate Object Manipulation. (a) Schematic diagram of the origami gripper integrated with a scissor structure and load cell for force measurement. (b) Sequence of gripper operation during force testing, showing states of approach, contact, force application (pressure reduction), and release (pressure increase), correlated with measured pressure and force over time. (c) Comparison of the gripper's configuration in the unactuated state (left) and at maximum actuation (right), indicating base displacement and reduction in jaw gap. (d) Time‐lapse images from Movie S1 and corresponding pressure profile demonstrating the gripper's ability to delicately grasp, move, and release a raspberry.

The actuator is integrated with two compliant mechanisms to create a soft gripper, which is shown schematically in Figure 1h. The axial contraction of the actuator closes the gripper jaws, allowing them to exert force. The grasping characterization and demonstration are depicted in Figure 4 and detailed in Figure S3. To quantify its performance, the grasping force is calculated from measurements taken by a load cell, which records the force transmitted through the mechanical scissor structure (Figure 4a,b). The grasping cycle is detailed in Figure 4b. Initially, the gripper approaches the test structure with no force applied (green region). Upon contact, an initial compressive force of approximately 0.22 N is registered (red region). As the internal pressure is reduced from 0 mbar to around −400 mbar, the gripping force increases from 0.15 to 0.19 N (yellow region). Subsequently, going back to atmospheric pressure reduces the applied force, causing the gripper to release (blue region). The gripper's range of motion is presented in Figure 4c, where a pressure change of −600 mbar displaces the actuator's base by 3.6 mm. This action reduces the initial gap between the gripper jaws from 11.0 to 7.8 mm.

The gripper's ability to handle delicate objects is demonstrated by grasping and manipulating a 4 g raspberry. (Figure 4d; and Movie S1). The process begins with the gripper approaching the fruit. The internal pressure is then reduced by −248 mbar to gently close the jaws and hold the raspberry (red region of the pressure curve). While maintaining a constant pressure, the gripper lifts and moves the raspberry upward and downward (yellow region). Finally, the fruit is returned to its original position, and the pressure is increased back to ambient, releasing the raspberry (blue region). The yellow arrows and blue dots indicate the raspberry being moved and hold, respectively.

To further demonstrate the versatility of the gripper, additional grasping experiments are performed using objects with different sizes and mechanical properties. Beyond the soft raspberry shown in Figure 4d, the gripper successfully grasps a jelly candy (4.3 g) and a rigid plastic syringe (4.0 g), demonstrating its ability to handle both compliant and rigid objects without mechanical reconfiguration or force control (Figure S7 and Movie S2).

In addition, the gripper is tested under submerged conditions using water as the actuation medium. A representative sequence of the underwater grasping is shown in Figure S7, with the corresponding behavior provided in Movie S3, which demonstrates the robustness of the monolithic origami actuator and its compatibility with operation in wet or confined environments.

## 3 Conclusions

In this work, we proposed a solution to address the critical challenges of sustainability and monolithic fabrication in soft robotics by developing a sustainable bio‐based resin derived from soybean oil with the suitable material properties to enable the fluidic actuation of soft robotic origami structures. With a composition of 90 wt.% renewable components, this formulation achieved a bio‐based carbon content of 73 %. The material characterization confirmed its suitability for functional applications in origami‐inspired soft robotics, revealing a Young's modulus of 18.9 MPa, an elongation at break of 19.6 %, and thermal as well as chemical robustness. This work thus directly addressed the field's heavy reliance on petroleum‐based polymers, by presenting a viable, environmentally responsible alternative for creating soft robotic systems.

Leveraging this custom resin, we demonstrated the monolithic, single‐step fabrication of a fluidically‐driven, origami‐inspired soft gripper using high‐resolution DLP. This method overcomes the limitations of traditional multi‐step, labor‐intensive assembly processes, utilizing compliance to generate mechanical work. The functional performance of our Kresling origami pattern actuator and integrated soft gripper was demonstrated through durability testing over 2000 cycles without failure and the successful capability of manipulating a diverse set of objects. This illustrates how the design of origami, which relies on folding rather than material stretching, can effectively enable the use of materials that have limited elongations compared to conventional silicone elastomers, thereby expanding their scope of application in soft robotics.

By co‐designing both the custom sustainable material and the soft robotic embodiment, we proposed a solution to bridge a critical gap in the field of soft robotics through DLP 3D printing using sustainable materials. Thus, this work represents a significant step toward environmentally responsible and scalable soft robotic systems. Looking forward, the high‐resolution 3D printing capability afforded by the developed resin opens promising possibilities for scaling down these concepts. We envision leveraging this technology to design and fabricate sustainable soft robots at the micrometer scale, enabling new applications in micromanipulation and biomedicine.

## 4 Experimental Section

### 4.1 Materials

Acrylated epoxidized soybean oil (AESO, M = 1160 g mol^−1^, average numbers of acryloyl and epoxide groups per molecule are 2.7 and 0.3, respectively [35].), tetrahydrofurfuryl methacrylate (THFMA), tetrahydrofurfuryl acrylate (THFA), pentaerythritol tetraacrylate (PETA), diphenyl(2,4,6‐trimethylbenzoyl)phosphine oxide (TPO), and propylene glycol monomethyl ether acetate (PGMEA) were purchased from Sigma Aldrich. 2‐(5‐Chloro‐2H‐benzotriazol‐2‐yl)−4‐methyl‐6‐di‐tert‐butylphenol (Tinuvin 326) was supplied by BASF. Genomer 4230 was kindly provided by RAHN GmbH (Germany). Acetone, isopropanol (IPA), ethanol, and dimethyl sulfoxide (DMSO) were purchased from Carl Roth GmbH. All reagents were used as received, without further purification.

### 4.2 Resin Preparation

The optimized resin was prepared by mixing AESO (75.0 wt.%), THFMA (15.0 wt.%), Genomer 4230 (9.5 wt.%), Tinuvin 326 (0.5 wt.%), and TPO (0.5 wt.% relative to the total mass of the other components) at room temperature under continuous magnetic stirring overnight to achieve a homogeneous solution. The prepared resin was stored at room temperature, protected from light, until use in 3D printing.

### 4.3 3D Printing

Monolithic origami‐inspired structures and test specimens were fabricated using an Asiga MAX X43 DLP 3D printer (Asiga, Australia). The device is equipped with a 405 nm UV LED, 43 µm pixel resolution, and 25 mW cm^−2^ light intensity. All samples were printed at room temperature with a layer thickness of 40 µm, an exposure time of 3 s, and a burn‐in layer exposure of 20 s. Printed samples were developed in an IPA bath for up to 1 min to remove unreacted resin without additional post‐curing. To enhance the adhesion of the printed samples to the print head, a non‐functionalized glass slide was affixed to the print head and used as the substrate.

### 4.4 UV–Vis Spectroscopy

The optical transparency of cured resin specimens was measured using a QEPro spectrometer (Ocean Optics, USA) with a DH‐2000‐BAL light source. The transmission measurement was carried out using UV‐cured specimens with different thicknesses. Samples with thicknesses of 60, 100, and 200 µm were prepared by sandwiching resin between glass slides separated by spacers of corresponding thickness and UV‐cured using a Lumatec Superlite S04 lamp. Bare glass slides were used as blanks.

### 4.5 Mechanical Characterizations

Tensile tests were conducted on vertically printed dog‐bone specimens (ISO 527‐2 type 5B) using a Universal Testing Machine Inspekt Table (Hegewald & Peschke GmbH, Germany) with a 1 kN load cell at room temperature. A strain rate of 1 mm s^−^^1^ was applied. Five replicates were measured to ensure reproducibility. The quasi‐static passive mechanical performance of the origami actuator structure was evaluated using a universal testing machine (ZwickRoell Z010); the finalized actuator, with an initial Kresling origami height of 33.14 mm, was subjected to a compressive deformation of 9.9 mm (30% of its initial height) at a constant rate of 0.1 mm s^−^^1^. The actuation performance in free stroke was determined from the relationship between internal pressure and axial displacement; a two‐stage rotary vane vacuum pump (model VC1621SG) was used to evacuate the actuator chamber while the resulting displacement was measured with a laser displacement sensor (Baumer OADK 25I7480/S14C) and a pressure sensor (Honeywell ABPDRRV600MGAA5). Regarding the gripper, the grasping force was quantified by actuating the device with the same vacuum setup. A mechanical scissor linkage transmitted the force from the jaws to a load cell (Futek LSB200) connected to a voltage amplifier (Omega TXDIN1600/S). Based on the linkage geometry, a mechanical advantage (ratio between output and input force) of 62 % was used to calculate the true grasping force from the measured data. In all experiments, analog data from the sensors were recorded using a data acquisition device (National Instruments DAQ‐USB 6212).

### 4.6 Thermogravimetric Analysis (TGA)

Thermal stability of printed specimen was assessed using an STA409C thermogravimetric analyzer (Netzsch, Germany) from room temperature to 600°C at a heating rate of 10°C min^−^^1^ under nitrogen atmosphere.

### 4.7 Swelling Test

To evaluate chemical resistance via swelling, 3D‐printed specimens with 7 mm diameter and 6 mm height were dried and weighed prior to testing. Samples were immersed in water, ethanol, IPA, PGMEA, DMSO, or acetone for up to 24 h. At predetermined intervals (1, 2, 3, 5, 8, and 24 h), specimens were removed, surface‐dried, and weighed. Mean values and standard deviations were calculated from three independent samples for each solvent.

### 4.8 Viscosity Measurement

Shear‐rate dependent viscosity of the resin was measured using an MCR 302 rheometer (Anton Paar, Germany) equipped with a CP‐50 measuring system. Measurements were performed at 20°C, 30°C, 40°C, and 50°C over a shear rate range of 1 to 100 s^−^^1^, with a sample volume of approximately 560 µL used for all tests.

### 4.9 Scanning Electron Microscopy

Printing resolution and microstructural analysis of printed specimens were performed using a Scios 2 HiVac Focused Ion Beam SEM (Thermo Fisher, USA). The gold sputtering of the samples was performed using the Cressington Sputter Coater 108 auto.

## Funding

This research was funded by the Deutsche Forschungsgemeinschaft (DFG, German Research Foundation) under Germany's Excellence Strategy – EXC‐2193/1 – 390951807. Additional financial support was provided by LyondellBasell through the FreiLyb Technology Center project (funding reference PSP 1100108301).

## Conflicts of Interest

The authors declare no conflicts of interest.

## Supporting information




**Supporting File 1**: advs73945‐sup‐0001‐SuppMat.docx.

**Supporting File 2**: advs73945‐sup‐0002‐MovieS1.mp4.

**Supporting File 3**: advs73945‐sup‐0003‐MovieS2.mp4.

**Supporting File 4**: advs73945‐sup‐0004‐MovieS3.mp4.

## Data Availability

The data that support the findings of this study are available from the corresponding author upon reasonable request.
