# Supplementary material for: Monolithic 3D Printing of Origami‐Inspired Soft Robotics from Sustainable Bio‐Based Resin
Source: Adv Sci (Weinh). 2026 Jan 21;13(15):e20529. doi: 10.1002/advs.202520529 (PMC13042658; doi:10.1002/advs.202520529)
Supplement: Supplementary file 1 — Supporting File 1: advs73945‐sup‐0001‐SuppMat.docx. [file ADVS-13-e20529-s002.docx]

**Supporting Information**

Supporting Information is available from the Wiley Online Library or from the author.

Supporting Information

Monolithic 3D printing of Origami-Inspired Soft Robotics from Sustainable Bio-Based Resin

Ramin Montazeri, Hugo de Souza Oliveira, Xin Li, Qingchuan Song, Bastian Rapp, Dorothea Helmer*, Edoardo Milana*

Close View of the Printed Structures


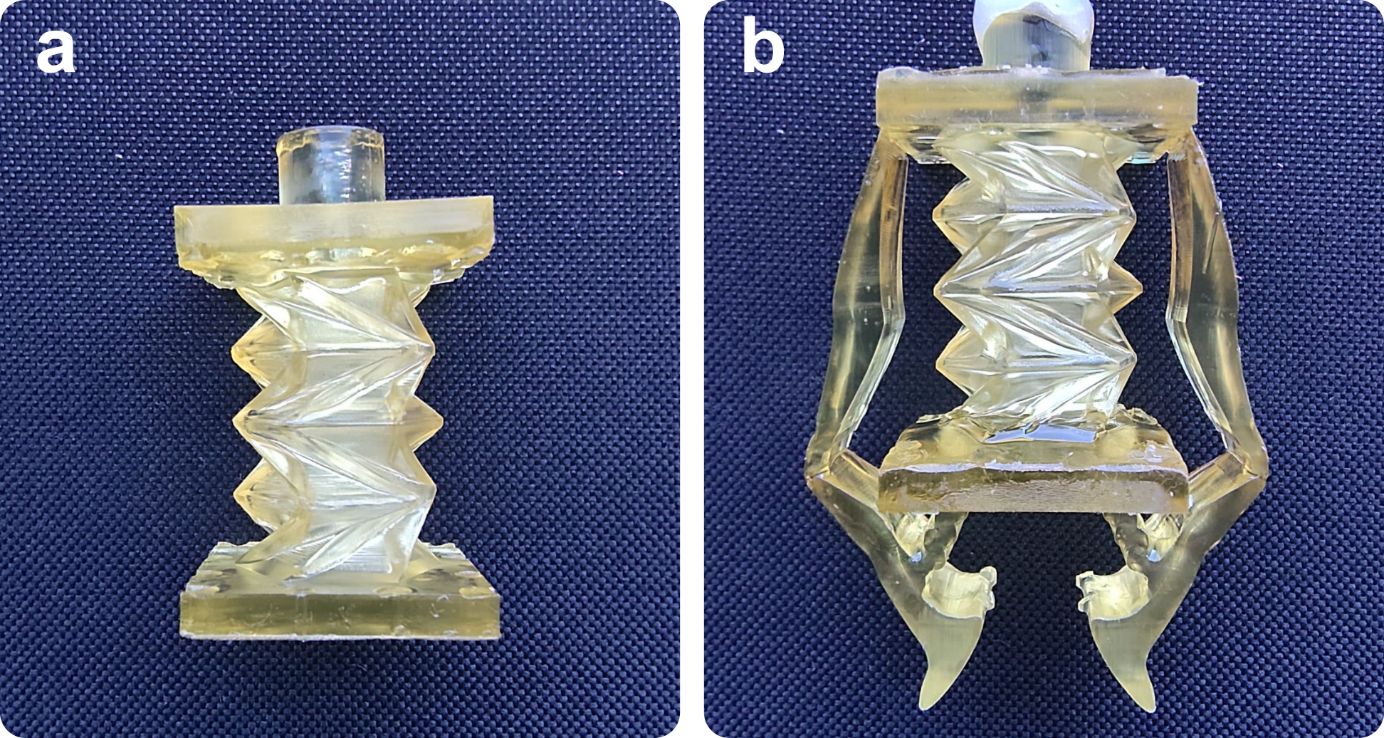


**Figure S1**. Printed Structures: a) Origami Actuator; b) Origami Actuator combined with the gripping mechanisms.

Experimental Setup

*
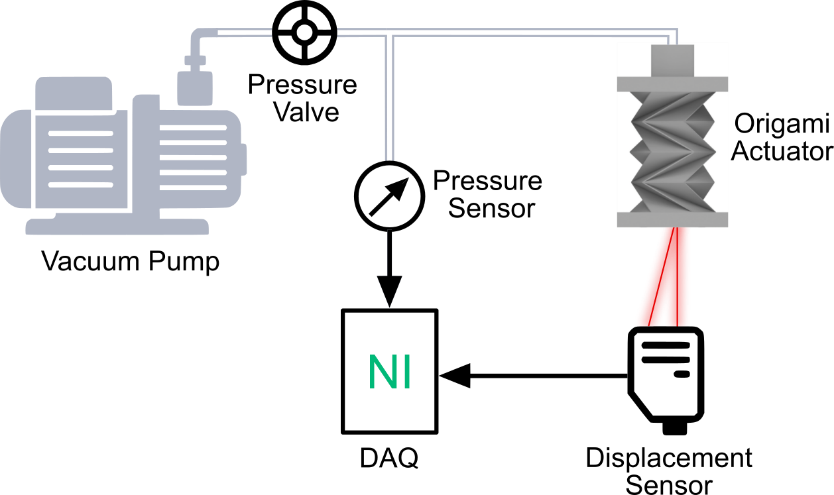
*

**Figure S2***.* Schematic of the experimental setup to measure the relation between pressure and displacement of the origami actuator

To characterize the relationship between the internal pressure and axial displacement of the origami actuator, we established the setup illustrated in Figure S2. The actuator was connected to a vacuum pump through a pressure regulation valve and a pressure sensor. The valve opening ratio was adjusted to control the applied negative pressure. A laser displacement sensor was positioned in front of the actuator to record its axial deformation. All sensor signals were simultaneously acquired using a National Instruments (NI) data acquisition (DAQ) system. The acquisition routine is demonstrated in Figure S4.


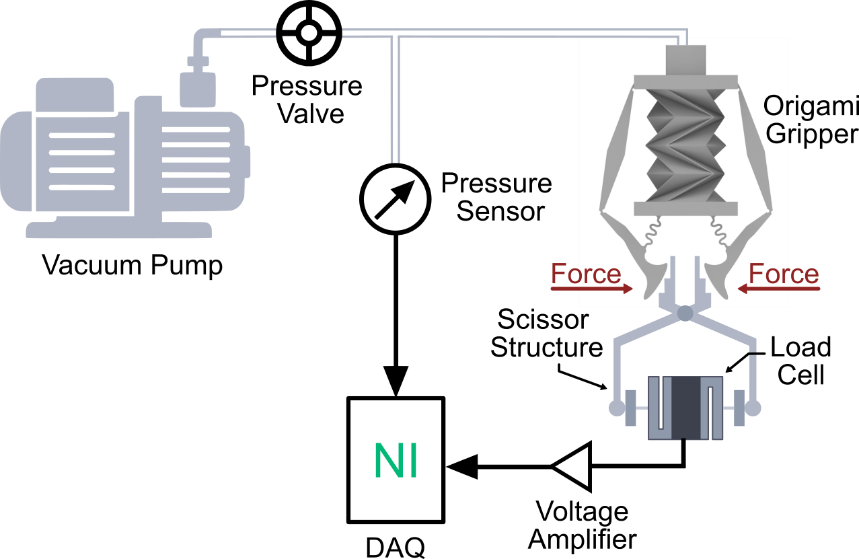


**Figure S3**. Schematic of the experimental setup to measure the relation between pressure and output force of the origami gripper

To investigate the relationship between the internal pressure and output force of the origami gripper, the setup shown in Figure S3 was employed. The gripper was connected to a vacuum pump through a pressure regulation valve and a pressure sensor. The applied negative pressure was controlled by adjusting the valve opening ratio. The gripping force was transmitted through a scissor-type mechanism and measured by a load cell. All sensor signals were synchronously acquired using a National Instruments (NI) data acquisition (DAQ) system. The acquisition routine is demonstrated in Figure S4.

LabVIEW Setup for Dual-Sensor Data Acquisition


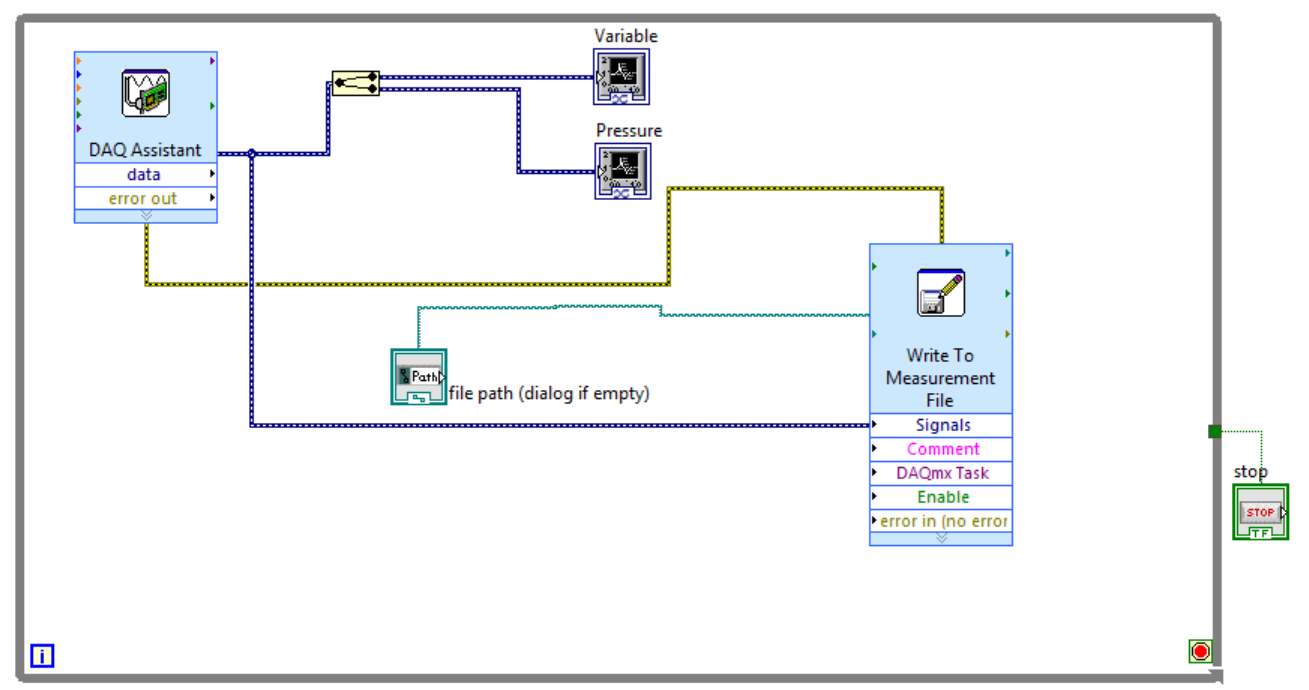


**Figure S4**. LabVIEW schematic used for simultaneous acquisition of pressure and displacement (or force) data.

The data acquisition routine was developed in LabVIEW to record synchronized signals from two analog input channels. The DAQ Assistant block initializes and controls the NI data acquisition hardware, continuously sampling both sensors in real time. Its outputs are routed to two display indicators: Pressure, showing the calibrated signal from the pressure transducer, and Variable, representing either the displacement (from a laser distance sensor) or the force (from a load cell), depending on the experimental configuration. A Path control specifies the output directory, and the Write to Measurement File block converts and stores the acquired data into a time-stamped LabVIEW Measurement (.lvm) file.

**Optimization of Resin Formulation**

**Table S1.** Bio-based resin formulations for DLP 3D printing. The resins are composed of acrylated epoxidized soybean oil (AESO) with either tetrahydrofurfuryl acrylate (THFA) or tetrahydrofurfuryl methacrylate (THFMA) as matrix components. THFA and THFMA act as bio-based reactive diluents, while pentaerythritol tetraacrylate (PETA) and Genomer 4230 serve as crosslinkers, Tinuvin 326 as UV absorber, and diphenyl(2,4,6-trimethylbenzoyl)phosphine oxide (TPO) as photoinitiator.

| Sample | AESO  [wt.%] | THFA  [wt.%] | THFMA  [wt.%] | PETA  [wt.%] | Genomer 4230  [wt.%] | Tinuvin 326  [wt.%] | TPO ^a)^  [wt.%] |
| --- | --- | --- | --- | --- | --- | --- | --- |
| 1 | 70 | 29.5 | 0 | 0 | 0 | 0.5 | 0.5 |
| 2 | 70 | 20 | 0 | 9.5 | 0 | 0.5 | 0.5 |
| 3 | 70 | 20 | 0 | 0 | 9.5 | 0.5 | 0.5 |
| 4 | 70 | 0 | 20 | 0 | 9.5 | 0.5 | 0.5 |
| 5 | 75 | 0 | 15 | 0 | 9.5 | 0.5 | 0.5 |
| ^a)^ The mass of TPO was calculated relative to the total mass of the other components. | | | | | | | |

**Quantitative Comparison of Key Metrics**

**Table S2.** Quantitative comparison of key metrics, including ultimate tensile strength, strain at break, Young’s modulus, maximum force, actuator pressure, cycle life, 3D printing resolution, and bio-based carbon content (BCC), where available, between the present study and two representative, closely related studies. The selected studies illustrate recent advances in sustainable DLP 3D printing of soft actuators, providing a basis for direct performance comparison.

| Metric | Present study | H. Li et al.^[23]^ | A. Ritere et al.^[19]^ |
| --- | --- | --- | --- |
| Sustainable Components | AESO, THFMA | Isobornyl acrylate | Acrylated rapeseed oil, Isobornyl acrylate |
| BCC of Optimized Resin [%] | 73 | N/A a) | 62 |
| Renewable Components [wt.%] | 90 | 60 | 70 |
| 3D Printing Resolution [µm] | 90 | 100 | 200 |
| Ultimate Tensile Strength [MPa] | 2.4 | 30 (at 25 °C) | 4.3 |
| Strain at Break [%] | 19.6 | 50 (at 25 °C) | 137 |
| Young’s Modulus [MPa] | 18.9 | 1060 (at 25 °C) | 3.6 |
| Cycle Life | 2000 | ND ^c)^ | ND |
| Origami-Inspired Structure | Yes | Yes | No |
| Origami FOLDING Force [N] | 1 | 399 (at 25 °C) ^b)^ | N/A |
| Gripping force [N] | 0.28 | N/A | ND |

^a)^ Not Applicable.

^b)^ Not Determined.

^c)^ Force required to fully open the folded origami structure.

**Leakage Analysis**

**
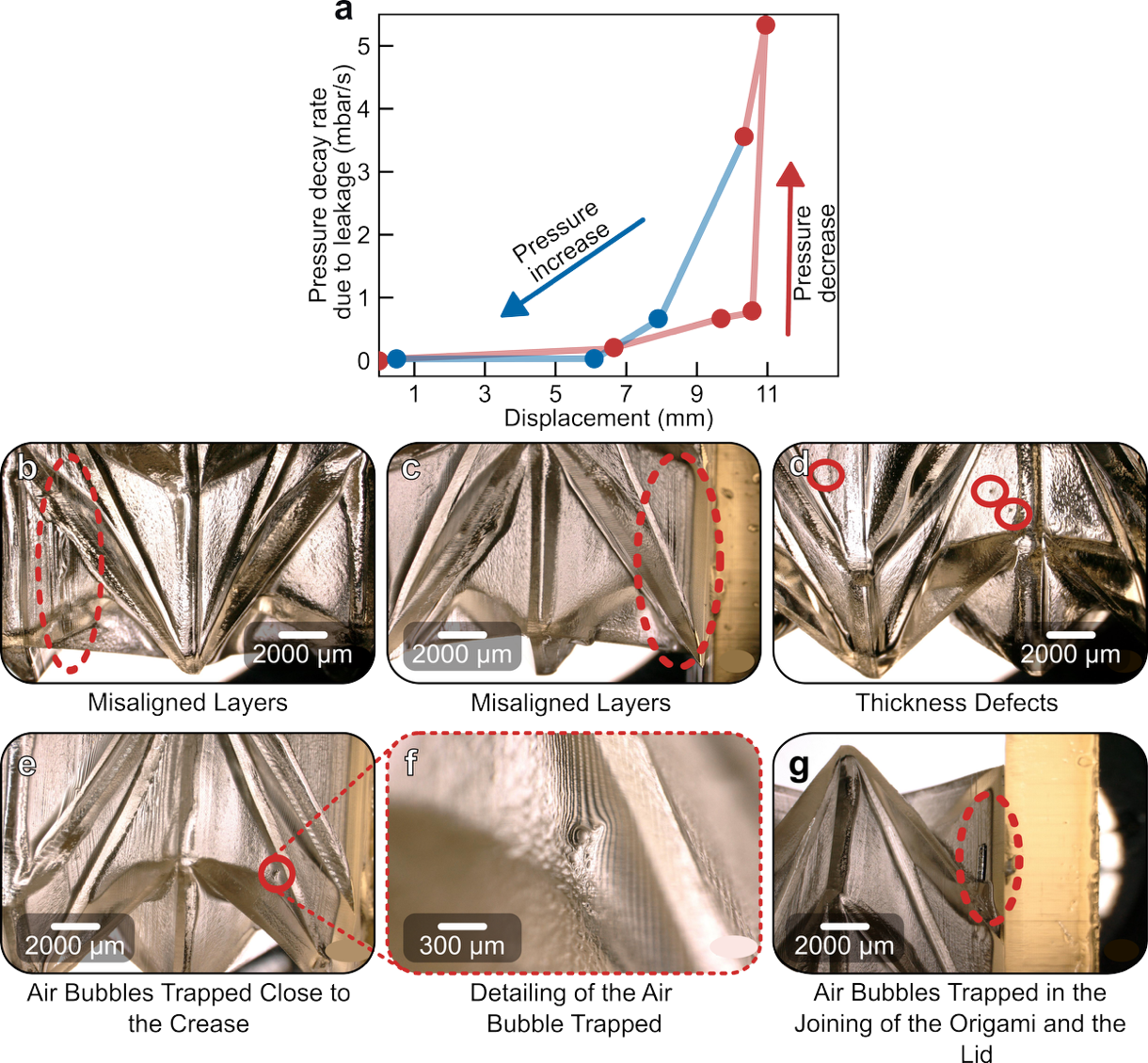
**

**Figure S5.** Quantitative leakage analysis and identification of potential leakage pathways in the origami actuator: (a) Pressure decay rate due to leakage (mbar/s) as a function of axial displacement, extracted from pressure–time segments at fixed valve setpoints. Leakage remains low at moderate deformations and increases sharply at larger axial displacements and higher pressure differentials. Arrows indicate pressure increase and decrease during actuation; (b) Optical micrograph showing misaligned layers on the left-hand side of the origami body, likely caused by transient layer adhesion to the vat during the DLP printing process; (c) Optical micrograph showing slight layer misalignment on the right-hand side of the origami body, attributed to delayed layer detachment and reattachment during printing; (d) Regions exhibiting reduced wall thickness compared to surrounding areas. Although fully printed and without open voids, these thickness defects may locally reduce mechanical strength; (e) Air bubble trapped between printed layers near a crease region on the right-hand side of the origami structure; (f) Magnified view of the air bubble highlighted in (e), showing subsurface air entrapment between layers; (g) Air bubble trapped within the body of the origami structure near the interface with the bottom base plate.

To quantify air leakage in the origami actuator, pressure–time data acquired during actuation experiments were analysed by extracting time intervals corresponding to fixed valve positions, where no active pressure control was applied. These intervals correspond to the yellow shaded regions in Figure 3b of the main manuscript. Under these quasi-static conditions, any observed pressure recovery reflects air leakage into the actuator chamber. The leakage rate was therefore defined as the temporal pressure recovery rate (mbar s⁻¹) and was obtained from linear fits to the pressure–time curves within each fixed-setpoint interval.

The resulting leakage rates are shown as a function of axial displacement in Figure S5a. For axial deformations below 10 mm and pressures above −100 mbar, the leakage rate remains low (approximately 0.25 mbar s⁻¹), consistent with stable actuator deformation. At larger deformations and more negative pressures, the leakage rate increases sharply, reaching values of approximately 5.2 mbar s⁻¹ close to the maximum deformation.

Figure S5b–g presents representative manufacturing-induced defects observed in the origami structure. These include layer misalignment, localized wall-thickness reduction, and trapped air bubbles within the printed material. Such defects primarily arise from resin flow limitations during the DLP printing process, particularly when using high-viscosity resins. During the layer-by-layer photopolymerization cycle, platform retraction and resin refill can induce transient vacuum conditions and localized shear, promoting incomplete resin redistribution and air entrapment.

Although these imperfections do not necessarily form fully open voids, they locally reduce wall thickness and introduce geometric discontinuities, which act as stress concentrators under compression or vacuum loading. As the internal pressure varies during actuation, these mechanically weakened regions are more susceptible to damage, providing preferential pathways for air infiltration and explaining the sharp increase in leakage rate observed at larger axial deformations.

**Crack Initiation and Propagation**


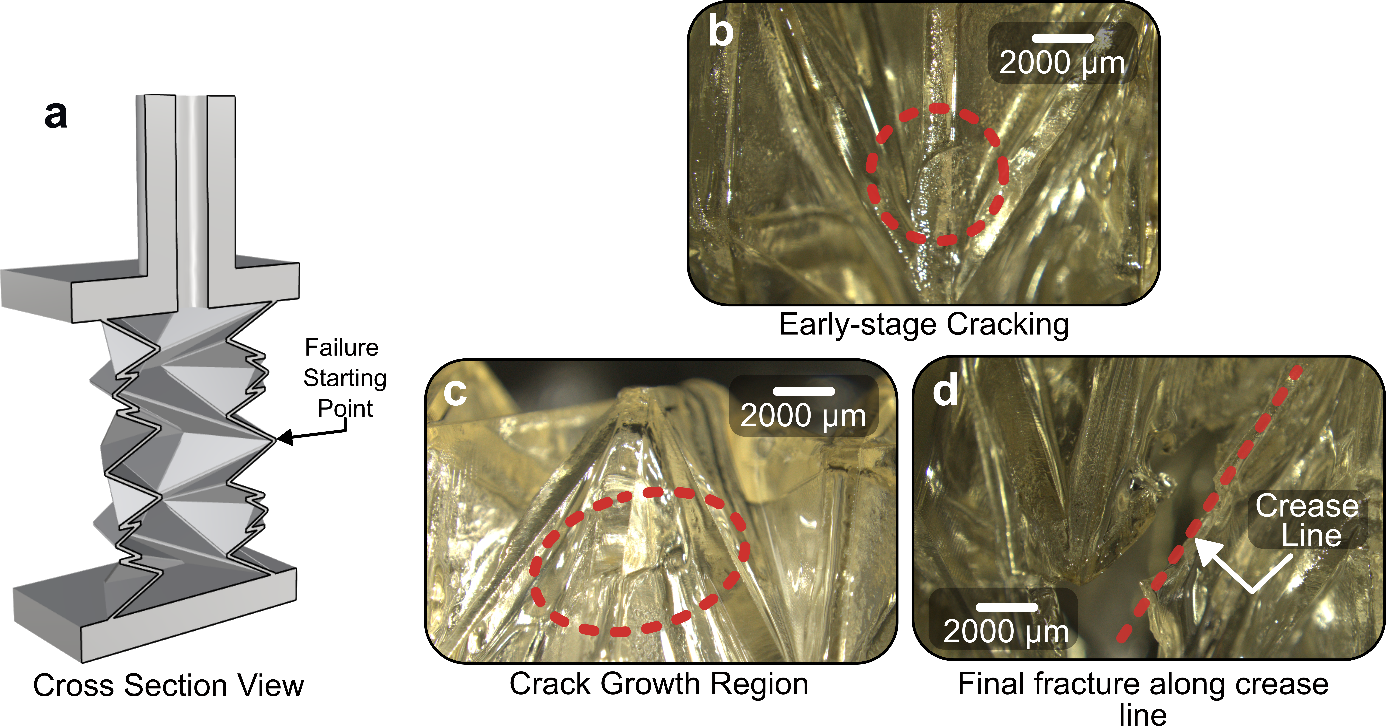


**Figure S6**. Crack initiation and propagation leading to structural failure of the origami actuator. (a) Schematic cross-sectional view of the origami actuator highlighting the region where micro-crack initiation is typically observed, located near the crease lines that experience localized stress concentration during cyclic deformation; (b) Optical micrograph showing early-stage micro-crack formation at the crease region; (c) Optical micrograph illustrating crack growth and propagation along the crease under continued deformation; (d) Optical micrograph showing final fracture occurring along the origami crease line after crack coalescence and growth.

Figure S6 illustrates the typical failure mechanism observed in the origami actuator under excessive deformation or prolonged cyclic loading. As schematically indicated in Figure S6a, micro-crack initiation preferentially occurs near the origami crease lines, where geometric discontinuities and localized bending lead to stress concentration. Optical micrographs (Figure S6b–d) show the progressive evolution of this damage, starting from early-stage micro-crack formation, followed by crack growth along the crease, and culminating in complete fracture of the structure.

**Additional Grasping Demonstrations and Submerged Operation**


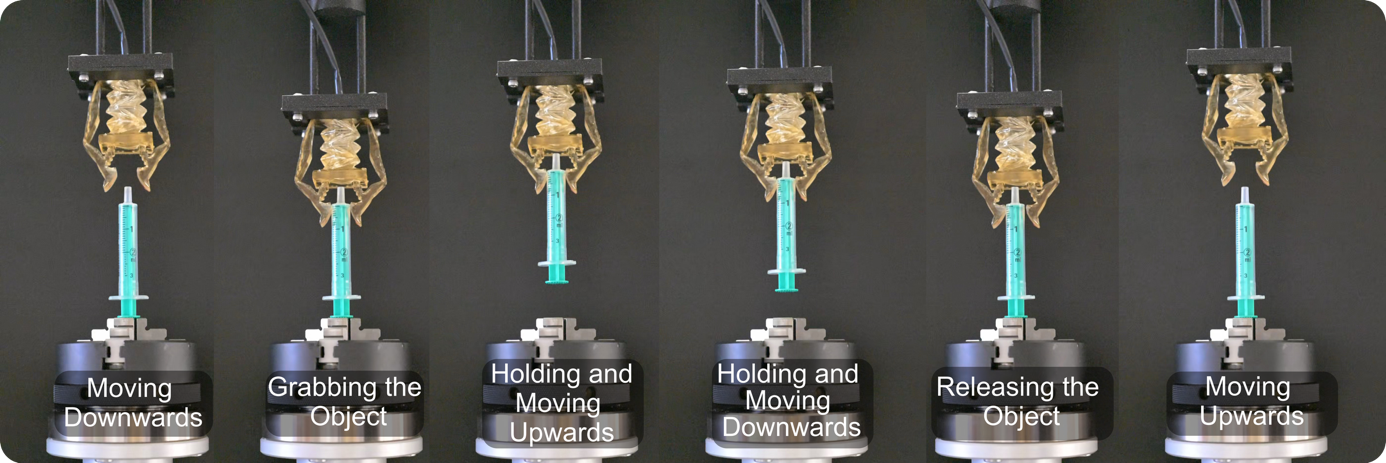


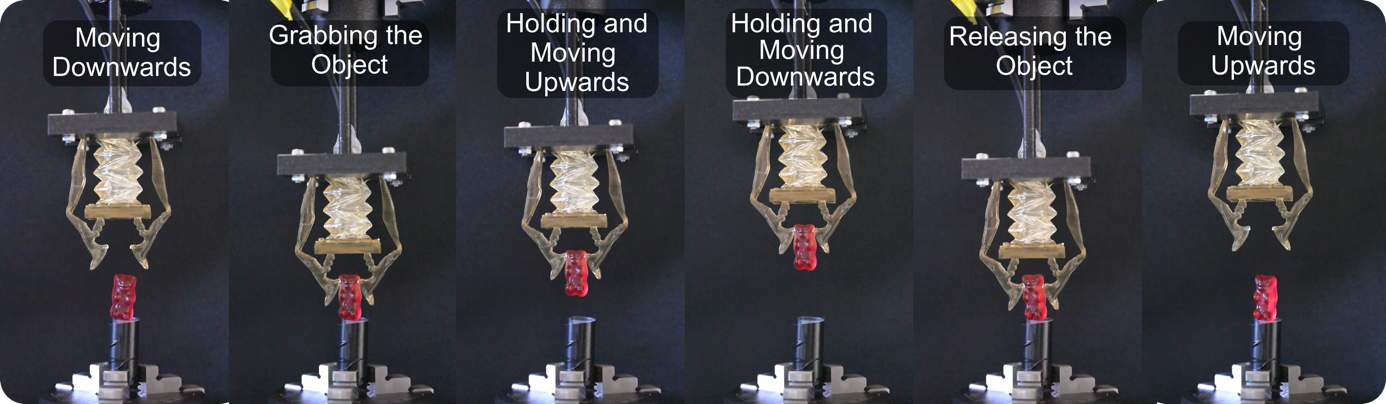


*
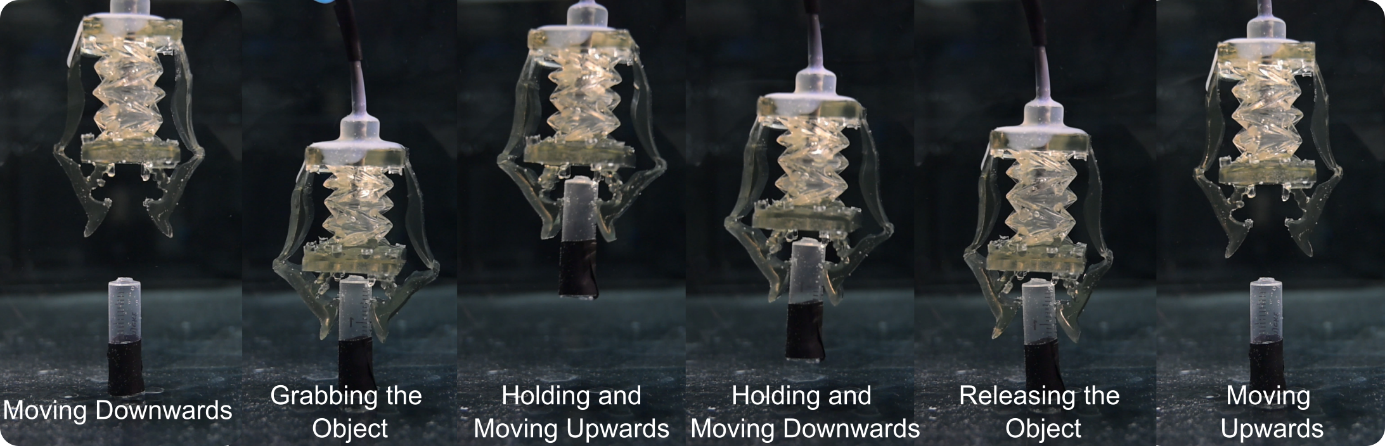
*

**Figure S7** Sequential images showing the operation of the monolithic origami-based soft gripper with a stiffer object (plastic syringe, top figure), an object with comparable stiffness (jelly bear, middle figure), and under submerged conditions using water as the actuation medium (bottom figure). The sequence illustrates the approach toward the object, grasping, lifting, controlled downward motion, release, and retraction. These demonstrations highlight the robustness of the origami actuator with objects of different stiffnesses and its compatibility with fluidic actuation in wet or confined environments. The corresponding dynamic behaviors are shown in Movie S2 and S3.

**Supplemental Movies**

**Movie S1.** Demonstration of the gripper's ability to delicately grasp, move, and release a raspberry (4.0 g).

**Movie S2.**

Demonstration of the adaptability of the monolithic origami-based soft gripper when grasping objects with different mechanical properties. The gripper successfully grasps and manipulates a jelly candy (4.3 g) with intermediate stiffness and a rigid plastic syringe (4.0 g) using the same actuator and without mechanical reconfiguration, illustrating its ability to handle both compliant and rigid objects.

**Movie S3.**

Demonstration of the monolithic origami-based soft gripper operating under submerged conditions using water as the actuation medium. The gripper approaches, grasps, lifts, and releases a rigid plastic object (5.5 g) while fully submerged, highlighting the robustness of the sealed origami actuator and its compatibility with fluidic actuation in wet or confined environments.
